# Identification of Lysosome‐Associated Protein Transmembrane‐4 as a Novel Therapeutic Target for Osteosarcoma Treatment

**DOI:** 10.1111/os.12692

**Published:** 2020-06-18

**Authors:** Zhe‐xiang Wang, Meng‐yang Guo, Jing Ren, Gui‐shi Li, Xu‐guo Sun

**Affiliations:** ^1^ School of Medical Laboratory Tianjin Medical University Tianjin China; ^2^ Precision Medicine Center Tianjin Medical University General Hospital Tianjin China; ^3^ Department of Joint Orthopaedics Yantai Yuhuangding Hospital Yantai China

**Keywords:** Invasion, LAPTM4B, Osteosarcoma, Proliferation, Therapeutic target

## Abstract

**Objective:**

The aim of the study is to evaluate the expression of lysosome‐associated protein transmembrane‐4 (LAPTM4B) in human osteosarcoma tissue samples collected in our hospital, and to explore the possible correlations between the clinical pathological features of osteosarcoma patients and LAPTM4B expression.

**Methods:**

Immunohistochemical (IHC) assays were performed to detect the expression levels of LAPTM4B in 62 tissue samples of osteosarcoma tissues and corresponding non‐tumor tissues. According to LAPTM4B staining intensity in tumor tissues, osteosarcoma patients were classified into LAPTM4B high expression and low expression groups. In addition, the potential correlations between LAPTM4B expression levels and clinical pathological features were evaluated. In addition, we detected the effects of LAPTM4B on the proliferation and invasion of esteosarcoma cells through colony formation assay and transwell assay, respectively. We further explored the potential effects of LAPTM4B on tumor growth and metastasis using *in vivo* animal model.

**Results:**

We revealed that LAPTM4B was highly expressed in human osteosarcoma tissues. We determined the significance between LAPTM4B and clinical features, including the tumor size (*P* = 0.004*) and the clinical stage (*P* = 0.035*) of osteosarcoma patients. Our results further demonstrated that ablation of LAPTM4B obviously blocked the proliferation and invasion of osteosarcoma cells *in vitro* and restrained tumor growth and metastasis in mice.

**Conclusion:**

We investigated the potential involvement of LAPTM4B in osteosarcoma progression and confirmed LAPTM4B as a novel therapeutic target for osteosarcoma.

## Introduction

Osteosarcoma (OC) is a common type of malignancy that originates in the bone, with high morbidity; it is the second leading cause of cancer‐related death among young people^1^, ^2^. Over the past 30 years, the combination of surgical resection and chemotherapy has been the most common treatment for advanced OC and has improved the 5‐year total survival rate of OC patients to approximately 70%^3^, ^4^, ^5^. However, due to chemotherapy resistance, nearly 40% of OC patients experience recurrence or metastases, which results in poor prognosis^6^, ^7^. Recently, targeted therapy became a promising therapeutic method for osteosarcoma, and multiple molecular targets have been identified, such as MYC or EGFR^8^, ^9^. To combat this malignancy more effectively, the development of additional therapeutic targets is urgently needed.

Lysosome‐associated protein transmembrane‐4 (LAPTM4B), which contains a lysosome localization motif and localizes on late lysosomes, is known as a member of the LAPTM family and is widely expressed in multiple human tissues^10^, ^11^. LAPTM4B was first cloned in human hepatocellular carcinoma (HCC) tissues in 2000 and could regulate autophagy and lysosome function^12^, ^13^. In addition, a previous study showed that LAPTM4B contributes to late endosomal ceramide export to regulate cell death pathways^14^. Another study demonstrated that LAPTM4B could activate the mTORC1 pathway through recruiting the LAT1‐4F2hc Leu transporter to lysosomes^15^.

The roles of LAPTM4B in the progression of multiple cancers has been widely discussed in in the literature^16^. LAPTM4B is overexpressed in several tumors, such as HCC, bladder cancer, and ovarian cancer^17^, ^18^, ^19^. Multiple studies have confirmed that LAPTM4B can affect cell proliferation, invasion, apoptosis, and autophagy of several types of cancer cells^11^, ^13^. LAPTM4B contributes to HCC growth and metastasis, which is regulated by AP4^20^. In breast cancer, LAPTM4B could predict lymph node metastasis and induce the aggressiveness of breast cancer cells^21^. Although LAPTM4B plays an important role in a variety of tumors, its potential effects on OC remains unclear. We assume that LAPTM4B is involved in OC, and that LAPTM4B might contribute to the proliferation and invasion of OS.

Interestingly, we found high expression levels of LAPTM4B in human osteosarcoma tissues and explored the potential correlations between LAPTM4B expression levels and clinical pathological characteristics (tumor size and clinical stage) of patients with osteosarcoma. We therefore found that LAPTM4B ablation remarkably blocked osteosarcoma cell proliferation and invasion and restrained tumor growth and metastasis in mice. LAPTM4B could, therefore, provide a novel and promising therapeutic target for osteosarcoma.

## Materials and Methods

### *Antibodies and Primers*

The antibodies used were: rabbit anti‐LAPTM4B (1:200 dilution for immunohistochemistry [IHC]; 1:1000 dilution for immunoblot, PA5‐43047, Thermo Fisher, Waltham, MA, USA) and mice anti‐β‐actin (1:2000 dilution, ab8227, Abcam, Cambridge, UK).

The quantitative polymerase chain reaction (PCR) primer sequences of LAPTM4B are as follows: forward, 5′‐ TGAACTGGGAGGTGACTTTGAG‐3′ and reverse, 5′‐CACACAGTTGCCCCCGTTTTTAC‐3′; The quantitative PCR primer sequences of GAPDH are as follows: 5′‐CGACCACTTTGTCAAGCTCA‐3′ and reverse, 5′‐GGTTGAGCACAGGGTACTTTATT‐3′.

Short hairpin RNA (shRNA) plasmids (Ready‐to‐package AAV) targeted LAPTM4B were bought from Addgene (Watertown, MA 02472, USA). The targeted sequences of the LAPTM4B shRNA plasmids were as follows: sense, 5′‐GGTCGCCTTCGGAGCGAAGGGTA‐3′.

### *Immunohistochemistry*

Tumor tissues were all collected from OC patients recruited in our hospital. To examine LAPTM4B expression in human osteosarcoma tissues, we conducted IHC assays. In brief, tumor or adjacent tissue sections were fixed with 4% paraformaldehyde (PFA) for 30 min at room temperature and subsequently blocked with 2% BSA for 20 min. Sections were then incubated with LAPTM4B antibodies at room temperature for 2 h. Subsequently, the sections were incubated with horseradish peroxidase (HRP) secondary antibody for 1.5 h, and diaminobenzidine was used as a chromogen substrate.

The expression level of LAPTM4B was scored as follows: staining intensity 0 = negative, 1 = low, 2 = modest, and 3 = high; and the proportion of stained cells 0 = 0% stained cells, 1 = 1%–30% stained cells, 2 = 31%–60% stained cells, and 3 = 61%–100% stained cells. A comprehensive score (score of staining intensity × score of stained cells percentage) <2 was considered negative staining, 2–3 low staining and >4 high staining. The sections of each patient were photographed within five visual fields, and two pathologists analyzed the sections.

### *Cell Culture and Transfection*

Human osteosarcoma cell lines: MG‐63 and U‐2 OS were bought from the ATCC, and maintained in EMEM and MMM (McCoy's 5a Medium Modified) culture medium, respectively, supplemented with 10% of fetal bovine serum at 37°C in a 5% CO_2_ incubator.

The shRNA plasmids were transfected into osteosarcoma cells using Lipofectamine 2000 (11668019, Invitrogen, CA, USA) according to the brochures. Stable knockdown clones were screened by lentivirus infection and used for the *in vivo* assays.

### *Quantitative Polymerase Chain Reaction Assay*

TRIzol (15596026, Invitrogen, CA, USA) was used to extract total RNA from tumor cells. Subsequently, the RNA was reverse‐transcribed by reverse transcriptase (M1701, Promega, Wisconsin, USA) to produce cDNA. Quantitative PCR was performed using a SYBR Ex Taq kit (638319, Takara, Japan), and the expression levels of LAPTM4B were normalized to the expression of GAPDH.

### *Immunoblot Assays*

The cancer cells or tissues were lysed by RIPA Buffer (9800, Cell Signaling, Danvers, MA). Then the total proteins were separated through SDS‐PAGE assays. The polyvinylidene fluoride (PVDF) membranes were blocked with 5% fat‐free milk in TBST buffer and incubated with the primary antibodies for the detection of LAPTM4B and β‐actin at room temperature for 2 h. Then the PVDF membranes were washed with TBST four times and the PVDF membranes were incubated with HRP‐conjugate secondary antibodies for 45 min. After washing, each blot was detected using an ECL kit. To analyze the intensity of each band, ImageJ software was used.

### *Colony Formation Assay*

A total of 1000 MG‐63 or U‐2 OS cells were seeded into a six‐well culture plate and transfected with control or LAPTM4B shRNA plasmids and maintained in a 37°C, 5% CO_2_ incubator for 24 h. After 2 weeks, cells were fixed with PFA for 20 min at room temperature, subsequently stained with 0.2% crystal violet for 20 min, and washed with phosphate‐buffered saline twice. Then the colony numbers were manually counted.

### *Transwell Assay*

Both MG‐63 and U‐2 OS cells were transfected with control or LAPTM4B shRNA plasmids for 48 h and then resuspended in serum‐free medium. The upper chambers of filters (8.0 μm membrane pores) were filled with 20% Matrigel in serum‐free medium and incubated at 37°C for 30 min. Approximately 10^5^ cells in 150 μL of medium were then seeded into the upper chambers of the inserts and induced to migrate toward the bottom chambers containing complete medium. After 24 h incubation, cells in the top chamber were removed using cotton swabs, and remaining cells were fixed with 4% PFA for 25 min and stained with 0.2% crystal violet for 20 min. Then the cell number was manually counted.

### *Tumor Growth Assay*

All *in vivo* assay processes were approved by our Institutional Animal Care and Use Committee. Briefly, MG‐63 cells were stably infected with control or LAPTM4B shRNA lentivirus. After infection for 48 h, approximately 5 × 10^5^ control or LAPTM4B‐depleted MG‐63 cells were subcutaneously implanted into athymic nude mice. After 2 weeks, mice were examined for tumors. The volume of each tumor was photographed and measured.

### *Tumor Metastasis Assay*

MG‐63 cells were infected with control or LAPTM4B shRNA lentivirus to stably knock down its expression. After 48 h, nearly 5 × 10^5^ infected cells were implanted into the tail vein of athymic nude mice. After 7 weeks, all metastasis tumor tissues were isolated from each group and photographed. and the metastasis degree was calculated and compared.

### *Statistics*

GraphPad 5.0 software was used in this study for statistical analysis. All data were represented as mean ± standard deviation (SD). The correlation between clinical pathological characteristics and protein levels were analyzed by χ^2^ analysis. Student's *t*‐test was used for statistical comparisons. * indicates *P* < 0.05 and statistical significance.

## Results

### *LAPTM4B is Enhanced in Human Osteosarcoma Tissues and Associated with the Clinico‐Pathological Features*

**Figure 1:**
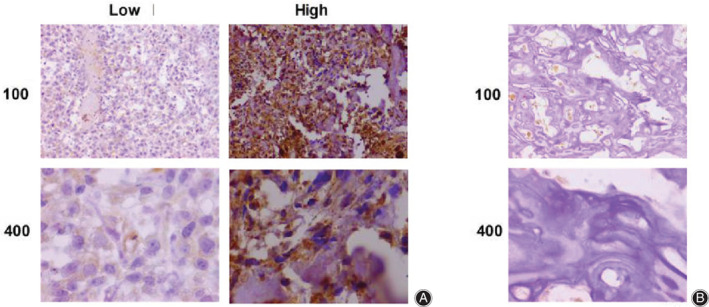
LAPTM4B was obviously highly expressed in osteosarcoma tissues from patients. (A) Immunohistochemistry (IHC) revealed LAPTM4B expression levels in osteosarcoma tissues and the representative images are exhibited (100× and 400× magnification, respectively). (B) IHC assays exhibited LAPTM4B expression levels in corresponding adjacent tissues and the representative images are shown (100× and 400× magnification, respectively).

To assess the possible involvement of LAPTM4B in the progression of osteosarcoma, 62 osteosarcoma tissue and the corresponding adjacent surgery samples were used to detect the expression of LAPTM4B by immunohistochemistry (IHC) assay. Notably, we found that LAPTM4B was mainly located in the cytoplasm of tumor cells and highly expressed in osteosarcoma tissues compared with adjacent non‐tumor tissues (Fig. 1A,B). Subsequently, tumor tissue samples were classified into two groups, LAPTM4B high‐expression and low‐expression groups, according to the staining intensity of LAPTM4B (Fig. 1A,B). We noticed that high expression of LAPTM4B was detected in 40/62 (64.5%) cases, while low expression of LAPTM4B accounted for 35.5% cases.

We then compared the significance of clinicopathological features between low and high expression LAPTM4B groups. The clinical features, such as patient age, patient gender, and differentiation, were recorded and analyzed, with the *P‐*values of 0.271, 0.055 and 0.176, respectively, suggesting no significance between low‐expression and high‐expression LAPTM4B groups in these features (Table 1). However, we found that the expression levels of LAPTM4B in tumor tissues was obviously correlated with tumor size and clinical stage, with the *P‐*values of 0.004* and 0.035*, respectively, suggesting a significant correlation between LAPTM4B expression and these clinical features.

**Table 1:** Relationships of LAPTM4B and clinicopathological characteristics in 66 patients with osteosarcoma

Feature	All *n* = 62	LAPTM4B expression		χ^*2*^	*P*
		Low	High		
		*n* = 22	*n* = 40		
Age (year)				1.213	0.271
< 25	28	12	16		
≥ 25	34	10	24		
Gender				3.674	0.055
Male	35	16	19		
Female	27	6	21		
Tumor size				8.417	0.004*
< 5 cm	27	15	12		
≥ 5 cm	35	7	28		
Differentiation				1.832	0.176
Low	24	11	13		
High	38	11	27		
Clinical stage				4.459	0.035*
I–II	40	18	22		
III	22	4	18		

In summary, we demonstrated that LAPTM4B was highly expressed in osteosarcoma tissues and correlated with the clinical pathological features.

### *Knockdown of LAPTM4B Obviously Restrained the Proliferation and Invasion of Osteosarcoma Cells* in vitro

**Figure 2:**
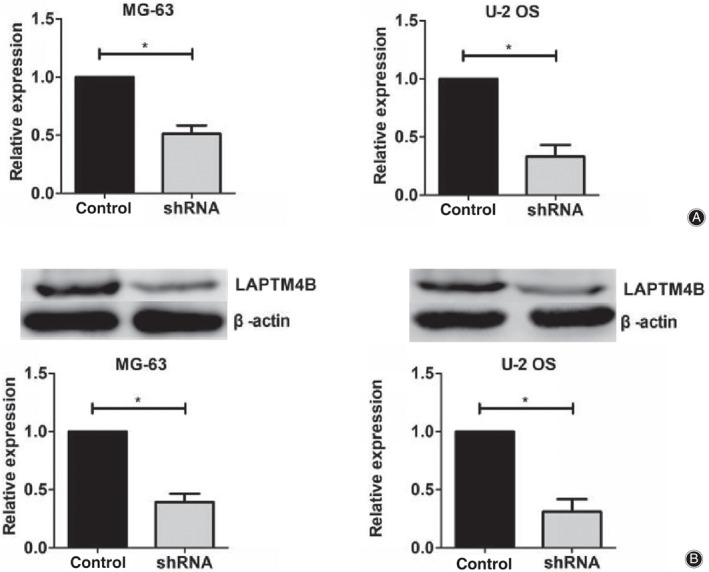
The expression levels of LAPTM4B were obviously decreased in both MG‐63 and U‐2 OS cells after the transfection of LAPTM4B‐targeted shRNA plasmids. (A) The results of quantitative PCR assays showed the obviously reduced expression levels of LAPTM4B in its short hairpin RNA (shRNA) plasmid‐transfected MG‐63 and U‐2 OS cells, respectively. (B) Immunoblot assays revealed the efficient decrease of LAPTM4B expression levels after the transfection of its shRNA plasmids in both MG‐63 and U‐2 OS cells. Results are presented as mean ± SD, *P < 0.05.

To further evaluate the potential involvement of LAPTM4B in osteosarcoma development, the expression of LAPTM4B was depleted by the transfection of its shRNA plasmids in two types of osteosarcoma cell lines: MG‐63 and U‐2 OS, respectively. The silence efficiency of LAPTM4B shRNA in these two types of cells was measured through quantitative PCR and western blot assays, respectively. Results indicated that the transfection of LAPTM4B shRNA plasmids obviously decreased the expression levels of LAPTM4B in MG‐63 and U‐2 OS cells (Fig. 2A,B).

**Figure 3:**
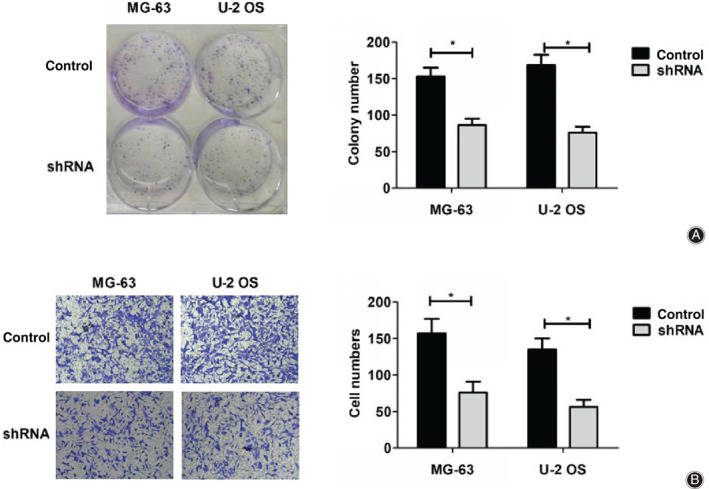
LAPTM4B regulates or affects and invasion of osteosarcoma cells *in vitro*. (A). Colony formation assays were performed using MG‐63 and U‐2 OS cells transfected with control or LAPTM4B short hairpin RNA (shRNA) plasmids, and colony numbers were manually counted (×1). (B). Transwell assays were conducted using both MG‐63 and U‐2 OS cells transfected with control or LAPTM4B shRNA plasmids, and the invasion degree was quantified by the numbers of stained cells (×200). Results are presented as mean ± SD, *P < 0.05.

We then explored the effects of LAPTM4B on osteosarcoma cell proliferation through colony formation assays. Notably, we found that the proliferation capacity was significantly blocked by LAPTM4B depletion, with obviously decreased cell numbers. (Fig. 3A).

Tumor cell invasion is required for cancer metastasis. Therefore, we sequentially detected the effects of LAPTM4B ablation on the invasion of osteosarcoma cells through transwell assays. Interestingly, MG‐63 and U‐2 OS cells exhibited a marked low invasive capacity through the Matrigel‐coated membranes when transfected with LAPTM4B shRNA plasmids, with significantly decreased cell numbers (Fig. 3B).

Therefore, these data revealed that LAPTM4B depletion dramatically suppressed cell proliferation and invasion of osteosarcoma cells *in vitro*.

### *LAPTM4B Facilitates Tumor Growth and Metastasis of Osteosarcoma Cells in Mice*

As we previously found, LAPTM4B affected the proliferation and invasion of osteosarcoma cells *in vivo*. To further explore the possible role of LAPTM4B in the progression of osteosarcoma, *in vivo* tumor growth and metastasis assays were performed.

**Figure 4:**
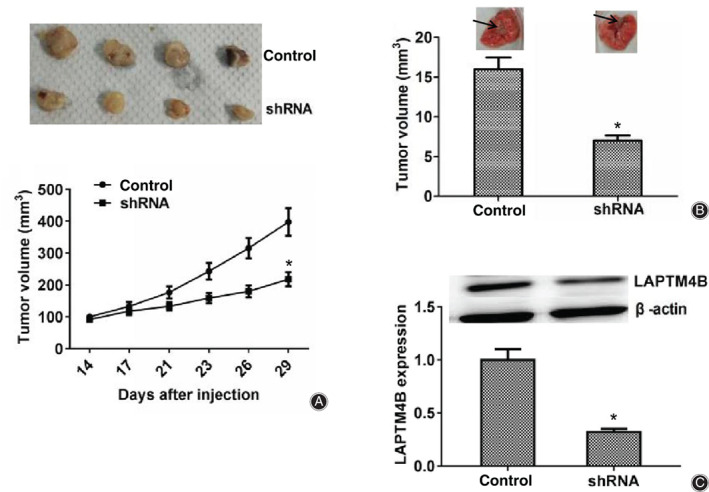
LAPTM4B contributes to tumor growth and metastasis of osteosarcoma cells in mice. (A) MG‐63 cells infected with control or LAPTM4B short hairpin RNA (shRNA) lentivirus were subcutaneously implanted into nude mice. After 2 weeks, tumors were isolated and photographed, and the volume of tumors was measured every week (*n* = 4 in each group). The tumor growth curves were calculated and analyzed according to the average volume of five tumors in between LAPTM4B knockdown and control groups. (B) MG‐63 cells infected with control or LAPTM4B shRNA lentivirus were sequentially implanted into the caudal vein of nude mice. Eight weeks later, tumors were isolated from mice and the metastasis volume was calculated in each group (*n* = 5 for each group). (C). Western blot assays were performed and exhibited the expression levels of LAPTM4B in control or knockdown group tumors isolated from mice. Results are presented as mean ± SD, *P < 0.05.

To detect tumor growth *in vivo*, MG‐63 cells were infected with control or LAPTM4B shRNA lentivirus for 48 h to stably deplete the expression of LAPTM4B, and subsequently injected into nude mice. After 2 weeks, tumors were formed and the volume of tumors in different groups was measured each week. Representative tumor images were photographed and are shown in Fig. 4A. Based on the images and tumor growth curves, we noticed that the tumor volume in the LAPTM4B ablation group was markedly smaller than that in the control group, (Fig. 4A). We further performed lung metastasis assays in mice to detect the effects of APTM4B on tumor metastasis. The results confirmed that the incidence of lung metastasis for LAPTM4B depletion MG‐63 cells was significantly decreased compared with the control (Fig. 4B). Then we detected the expression levels of LAPTM4B in tumor tissues from mice, and western blot assays showed that the expression of LAPTM4B in knockdown groups was obviously reduced compared with the control (Fig. 4C). In conclusion, all these results confirmed that LAPTM4B has a critical role in the growth and metastasis of osteosarcoma.

## Discussion

Due to its fast growth and early metastasis, osteosarcoma has a high mortality rate among young people^5^. Up to now, the pathogenesis and tumor driver genes of osteosarcoma have not been clarified, and the main treatment for osteosarcoma is surgery, chemotherapy, and adjuvant chemotherapy^22^. However, 40% of patients with osteosarcoma are at risk of pulmonary metastasis and recurrence, with a 5‐year survival rate of only 20%^23^, ^24^. Therefore, target therapy is a promising clinical treatment for osteosarcoma^25^. Notably, we here identified that a member of the LAPTM family, LAPTM4B, has the potential to become a therapeutic target for osteosarcoma. We found high expression of LAPTM4B in 62 human osteosarcoma tissues. Moreover, our data revealed that LAPTM4B expression was markedly associated with the clinical‐pathological features, including tumor size (*P* = 0.004*) and clinical stage (*P* = 0.035*), of patients with osteosarcoma.

Performing colony formation assays, we found that the proliferation of osteosarcoma cells was dramatically inhibited as a result of LAPTM4B shRNA transfection. Similarly, LAPTM4B knockdown suppressed the proliferation of lung adenocarcinoma cells through the PI3K/AKT signal pathway^26^. In hepatocellular carcinoma (HCC), LAPTM4B also facilitates cell proliferation and tumor growth *via* AKT signaling pathways^20^. We therefore hypothesize that LAPTM4B regulates osteosarcoma cell proliferation in a similar way. Our data further confirmed through transwell assays and lung metastasis assays, respectively, that LAPTM4B regulated cell invasion and tumor metastasis of osteosarcoma. In breast cancer and HCC, LAPTM4B also contributes to tumor metastasis and cell aggressiveness, and is associated with clinical features such as lymph node metastasis^21^, ^27^. Previous studies have also indicated that the high expression of LAPTM4B is a risk factor for recurrence and is associated with poor prognosis in non‐small‐cell lung cancer^16^, ^28^. In addition, the ablation of LAPTM4B suppressed the proliferation, invasion, and angiogenesis of HeLa cells^29^. LAPTM4B could activate the EGFR signal pathway to promote the development of gastric cancer, which was repressed by Beclin1^30^. These studies, together with our findings, suggested LAPTM4B as a novel and promising therapeutic target for the treatment of multiple cancers. In addition to its regulatory function in a variety of tumors, LAPTM4B also has several important physiological functions in non‐tumor cells. LAPTM4B mediates amino acid transporter interaction and late endosomal ceramide export, and hence regulates cell death pathways^14^, ^31^. In addition, LAPTM4B could lead to uptake of Leu into lysosomes and activate the mTORC1 through recruiting LAT1‐4F2hc to lysosomes^15^. We found that LAPTM4B was correlated with the clinical features and could promote the progression of osteosarcoma, although the mechanism remains unclear. Given the complexity of the LAPTM4B function, we should next explore whether LAPTM4B could regulate osteosarcoma development in a different manner, such as a lysosome‐dependent manner.

### *Conclusion*

We here revealed the high expression of LAPTM4B in human osteosarcoma tissues, and its expression level was obviously correlated with the clinical pathological features (including tumor size and clinical stage) of patients who underwent osteosarcoma. Ablation of LAPTM4B led to the inhibition of cell proliferation and invasion *in vitro*. In addition, LAPTM4B facilitates the growth and metastasis of osteosarcoma cells in mice. These findings therefore suggest LAPTM4B as a novel and promising therapeutic target for osteosarcoma in the future.
